# Innovative cropping systems designed to reach both environmental and production targets: Data set of biotic and abiotic variables from a twelve-year French field trial

**DOI:** 10.1016/j.dib.2024.110398

**Published:** 2024-04-05

**Authors:** Caroline Colnenne-David, Marie-Hélène Jeuffroy, Gilles Grandeau, Fabien Ferchaud, Thierry Doré

**Affiliations:** a Université Paris-Saclay, AgroParisTech, INRAE, UMR Agronomie, Palaiseau 91123, France; b BioEcoAgro Joint Research Unit, INRAE, Université de Liège, Université de Lille, Université de Picardie Jules Verne, Barenton-Bugny 02000, France; c Université Montpellier, CIRAD, INRAE, IRD, InstitutAgro Montpellier, UMR Eco&Sols, Montpellier 34060, France

**Keywords:** Long-term field trial, Crop growth, Crop biomass, Yield, Soil physicochemical properties, Energy consumption, Treatment frequency index, Agricultural practices

## Abstract

The data set describes variables collected from a French (N 48.84°, E 1.95°) field trial, over a twelve-year period (2009-2020), in which four innovative cropping systems designed to reach multiple environmental and production goals were assessed. The four cropping systems were designed with new combinations of agricultural practices; they differed in terms of pesticide uses, nitrogen inputs, tillage practices, and crop sequences. Both biotic and abiotic variables were measured. In a previous data paper, we focused on nitrogen fluxes collected from two systems, over eight years (2009-2016). In the present one, we enlarge the scope of the variables, including more crop descriptions and environmental indicators, from all four systems, and over a longer period (2009-2020). The biotic data are: growth stages; aboveground plant nitrogen content and biomass collected at different growth stages, depending on the species; yield components of all the crops; and yield harvested with a combine machine. No weed, crop disease, and pest data are described. The abiotic data are physical and chemical properties of the soil (i.e. texture, calcium carbonate content, pH, organic carbon contents, and nitrogen contents) collected at different assessment periods. All agricultural practices, and climate were regularly recorded, and the treatment frequency indexes and the energy consumptions were computed. These data could be used for benchmarking, to design low-input systems, to improve models for parameterization and validation, and to increase the predictive accuracy of models of crop growth and development, specifically for orphan species such as linseed, faba bean or hemp, and for soil carbon and soil nitrogen fluxes in various conditions.

Specification Table

**Table:** 

Subject	Agronomy and Crop Science
Specific subject area	Cropping systems: no-pesticide, low energy, low greenhouse gas, low input. Crop descriptions, soil properties, agricultural practices, from field-trial, over 2009-2020.
Data format	Raw, Analyzed, Computed All the file layouts are “.csv” files, making data readily available for all users.
Type of data	Tables, Figure
Data collection	Most of the crop and soil samples were collected manually from the trial. Aerial plant nitrogen contents were analyzed by the Dumas combustion method in the laboratory, after plants had been oven-dried at 80°C for 48 hours. Soil properties (texture, carbon content, calcium carbonate content, nitrogen content, pH) were analyzed with the requisite international standard methods in the laboratory. Yields were harvested with a combine machine. Agricultural practices were recorded from the field trial over all the assessment. Treatment frequency indexes were calculated according to [1]. Energy consumptions were based on [2]. Climatic data were collected from an automated meteorological station near the trial (150 m).
Data source location	France, N 48.84°, E 1.95°
Data accessibility	Open Research Data Portal at INRAE. Under the Etalab Open License 2.0, compatible CC-BY 2.0 https://doi.org/10.57745/5TJJZA
Related research article	C. Colnenne-David, G. Grandeau, M-H. Jeuffroy, T. Doré, Ambitious multiple goals for the future of agriculture are unequally achieved by innovative cropping systems, Field Crop Res. 210 (2017) 114-128, 10.1016/j.fcr.2017.05.009.

## 1 Value of Data

• The data were collected from four innovative cropping systems, designed with multiple environmental and production objectives, assessed in a wide (6.2 ha) and long-term (2009-2020) field experiment in France (N 48.84°, E 1.95°).
• The data were used to assess the environmental and production performances of the systems [3,4,5,6], and to write a previous data paper [7]. Here, the data cover the entire assessment period of the cropping systems, and encompass a wider range of variables.
• These data provide ongoing research material for benchmarking, to design new systems that eliminate pesticide use and decrease energy consumption, nitrogen losses, and greenhouse gas emissions, in northern Europe.
• The data could be used to improve models of plant growth, soil nitrogen fluxes, and soil carbon sequestration, and to enhance their predictive accuracy.
• The data can also be used to calculate new indicators based on nitrogen flux, and soil carbon sequestration measurements.

## 2 Background

The aim of the “Innovative Cropping systems under Constraints” project was to design four innovative cropping systems, combining multiple environmental and production objectives to deliver ecosystem services [3], and to assess them in a wide (6.2 ha) and long-term (2009-2020) field trial in France (N 48.84°, E 1.95°) [4,5], and [6].

The objective of this data paper is to pull together the highest number of data collected from this experiment. Compared to the previous data paper driven from the same project [7], we added a wide range of variables (i.e. growth stages, yield components, soil carbon contents), and environmental indicators (i.e. treatment frequency indexes, energy consumption). Data were provided from all four systems, and the collection period was longer (twelve years) than that presented previously (seven years), for all the variables including those already gathered in [7]. No data on weeds, crop diseases, or pests are provided in this paper. Due to the COVID pandemic in 2020, no agricultural practice over the spring period was allowed, resulting in low yields, and all measurements were banned. Despite such unusual cropping system management, some data were delivered to further understanding relating to the soil carbon content measured in October 2020.

## 3 Data Description

We classified the data in five groups: (1) plant measurements; (2) soil properties; (3) agricultural practices; (4) environmental indicator results; and (5) climate. The four cropping systems are denoted as follows: productive with high environmental performances (PHEP); no pesticide use (No-Pest); low energy consumption (L-EN); and low greenhouse gas emissions (L-GHG). In all the files, MD stands for missing data.

### 3.1 Plant measurements

#### 3.1.1 File: data_growth_stage_2023

This file includes data of growth stages of all species, measured regularly in the four cropping systems over the 2009-2020 period. There are nine columns: (1) year of harvest (YYYY); (2) name of the cropping system (CS): productive with high environmental performances (PHEP), no pesticide use (No-Pest), low energy consumption (L-EN), and low greenhouse gas emissions (L-GHG); (3) number of replicate (1 to 3); (4) number of plot (1 to 12); (5) species (for precise meanings see file “glossary_species”); (6) date of measurement (DD/MM/YYYY); (7) growth stage (for precise meanings see file “glossary_growth_stage”); (8) code (for each species see file “growth scales”); and (9) comments.

#### 3.1.2 File: glossary_species_2023

This file contains meanings of species abbreviations. There are two columns: (1) abbreviation; and (2) meaning of the abbreviation.

#### 3.1.3 File: glossary_growth_stage_2023

This file includes meanings of growth stage abbreviations. There are three columns: (1) growth stage abbreviation; (2) meaning of the abbreviation; and (3) code of growth stage.

#### 3.1.4 File: data_plant_biomass_nitrogen_content_2023

This file contains data of the aboveground dry biomass, and N content, of the crops measured at different growth periods, in the four cropping systems, over the 2009-2020 period. There are eighteen columns: (1) year of harvest (YYYY); (2) name of cropping system (productive with high environmental performances, PHEP; no pesticide use, No-Pest; low energy consumption, L-EN; and low greenhouse gas emissions, L-GHG); (3) number of replicate (1 to 3); (4) number of plot (1 to 12); (5) number of sample (1 to 12); (6) surface of the sample (m²); (7) species (for precise meanings see file “glossary_species”); (8) cover crop species (for precise meanings see file “glossary_species”); (9) date of measurement (DD/MM/YYYY); (10) growth stage (for precise meanings see file “glossary_growth_stage”); (11) organ measured; (12) biomass (gram) of the sample (0% of moisture); (13) biomass of the sample (g.m^−2^; 0% of moisture); (14) biomass of the sample (t.ha^−1^; 0% of moisture); (15) number of samples used for nitrogen (N) content analyses (1 and 2; 3 and 4; 5 and 6,…); (16) nitrogen (N) content of the sample (kg N.q^−1^ of dried matter); (17) quantity of nitrogen (N) in the aboveground biomass (g N.m^−2^); and (18) quantity of nitrogen (N) in the aboveground biomass (kg N.ha^−1^).

#### 3.1.5 Chapter: yield components

##### 3.1.5.1 File: data_yield_component_cereals_2023

This file includes data relating to the yield components of cereals (barley, oat, triticale, wheat), sown in the four systems, over the 2009-2020 period. There are fifteen columns: (1) year of harvest (YYYY); (2) name of cropping system (productive with high environmental performances, PHEP; no pesticide use, No-Pest; low energy consumption, L-EN; and low greenhouse gas emissions, L-GHG); (3) number of replicate (1 to 3); (4) number of plot (1 to 12); (5) number of the sample (1 to 12; 1_0N to 3_0N; some peculiarities); (6) surface of sample (m²); (7) species (for precise meanings see file “glossary_species”); (8) date of the measurement (DD/MM/YYYY); (9) number of plants per sample; (10) number of plants per sample at the beginning of tillering; (11) number of plants per sample counted over spring period; (12) average number of tillers with more than three sub-tillers; (13) total number of ears per sample; (14) total kernel biomass (gram) per sample (0% of dry matter); and (15) total number of kernels per sample.

##### 3.1.5.2 File: data_yield_component_legumes_2023

This file contains data on the yield components of legumes (faba bean, pea, soybean), sown in the four systems, over the 2009-2020 period. There are sixteen columns: (1) year of harvest (YYYY); (2) name of cropping system (productive with high environmental performances, PHEP; no pesticide use, No-Pest; low energy consumption, L-EN; and low greenhouse gas emissions, L-GHG); (3) number of replicate (1 to 3); (4) number of plot (1 to 12); (5) number of the sample (1 to 12); (6) surface of sample (m²); (7) species (for precise meanings see file “glossary_species”); (8) date of the measurement (DD/MM/YYYY); (9) number of plants per sample; (10) total number of branches per sample; (11) total number of fertile pods per sample; (12)) total number of sterile pods per sample; (13) total number of pods per sample; (14) total kernel biomass (gram) of the sample (0% of dry matter); (15) total number of kernels per sample; and (16) thousand-kernels weight (gram) of the sample (0% of dry matter).

##### 3.1.5.3 File: data_yield_component_linseed_2023

This file includes data relating to the yield components of linseed, only sown in the low energy consumption system, over the 2009-2014 period. There are nineteen columns: (1) year of harvest (YYYY); (2) name of cropping system (low energy consumption, L-EN); (3) number of replicate (1 to 3); (4) number of plot (3, 5, and 12); (5) number of the sample (1 to 12; 1_0N to 3_0N); (6) surface of sample (m²); (7) species (for precise meanings see file “glossary_species”); (8) date of the measurement (DD/MM/YYYY); (9) number of plants per sample; (10) total number of branches per sample; (11) total number of fertile branches per sample; (12) total number of sterile branches per sample; (13) total number of capsules per sample; (14) total number of fertile capsules per sample; (15) total number of sterile capsules per sample; (16) total number of sterile peduncles per sample; (17) total kernel biomass (gram) of the sample (0% of dry matter); (18) total number of kernels per sample; and (19) thousand-kernels weight (gram) of the sample (0% of dry matter).

##### 3.1.5.4 File: data_yield_component_maize_2023

This file contains data of the yield components of maize, only sown in the no pesticide use system and the low greenhouse gas emissions system, over the 2009-2020 period. There are thirteen columns: (1) year of harvest (YYYY); (2) name of cropping system (no pesticide use, No-Pest; and low greenhouse gas emissions, L-GHG); (3) number of replicate (1 to 3); (4) number of plot (2, 4, 6, 8, 9, and 11); (5) number of the sample (1 to 15; 1_0N to 3_0N); (6) surface of sample (m²); (7) date of the measurement (DD/MM/YYYY); (8) number of plants per sample; (9) total number of cobs per sample; (10) total number of fertile cobs per sample; (11) average of rows per cob in the sample; (12) total kernel biomass (gram) of the sample (0% of dry matter); and (13) thousand-kernels weight (gram) of the sample (0% of dry matter).

##### 3.1.5.5 File: data_yield_component_rape_2023

This file includes data relating to the yield components of winter rape, sown in the productive with high environmental performances system, the low energy consumption system, and the low greenhouse gas emissions system, over the 2009-2020 period. There are fourteen columns: (1) year of harvest (YYYY); (2) name of cropping system (productive with high environmental performances, PHEP; low energy consumption, L-EN; and low greenhouse gas emissions, L-GHG); (3) number of replicate (1 to 3); (4) number of plot (1, 3, 4, 5, 6, 7, 9, 10, and 12); (5) number of the sample (1 to 12; 1_0N to 3_0N); (6) surface of sample (m²); (7) date of the measurement (DD/MM/YYYY); (8) number of plants per sample; (9) total number of branches per sample; (10) total number of fertile pods per sample; (11) total number of short peduncles (corresponding to sterile pods) on the branches in the sample; (12) total number of long peduncles (corresponding to sterile pods) on the branches in the sample; (13) total number of burst pods (sterile pods) in the sample; and (14) total potential number of pods (both fertile and sterile) in the sample.

#### 3.1.6 File: data_yield_combine_all_species_2023

This file contains data of yields for all species, collected on each plot in the four cropping systems, each year over the 2009-2020 period. There are eleven columns: (1) year of harvest (YYYY); (2) name of the cropping system (productive with high environmental performances, PHEP; no pesticide use, No-Pest; low energy consumption, L-EN; and low greenhouse gas emissions, L-GHG); (3) number of replicate (1 to 3); (4) number of plot (1 to 12); (5) number of the sample; (6) species (for precise meanings see file “glossary_species”); (7) date of measurement (DD/MM/YYYY); (8) surface area of the sample (expressed in m²); (9) moisture of the sample expressed in percent of dry biomass (((wet soil mass - dry soil mass)/ dry soil mass)*100); (10) thousand-kernels weight (0% of dry matter); and (11) yield of the sample (for all species except hemp: expressed in q.ha^−1^ per hectare, 0% of dry matter; for hemp: expressed in tons of dry matter per hectare, 0% of dry matter).

### 3.2 Soil properties

#### 3.2.1 File: data_soil_physic_chemic_2023

This file includes data on soil texture and some soil chemical variables, measured at 0-25 cm, collected at the same time, from the twelve plots, at the beginning of the trial implementation (in 2009). There are fourteen columns: (1) date of sampling (DD/MM/YYYY); (2) name of the cropping system (productive with high environmental performances, PHEP; no pesticide use, No-Pest; low energy consumption, L-EN; and low greenhouse gas emissions, L-GHG); (3) number of replicate (1 to 3); (4) number of plot (1 to 12); (5) clay content (g.kg^−1^); (6) fine silt content (g.kg^−1^); (7) coarse silt content (g.kg^−1^); (8) fine sand content (g.kg^−1^); (9) coarse sand content (g.kg^−1^); (10) organic carbon (Corg) content (Corg: g.kg^−1^); (11) mineral carbon (Cmin) content (Cmin: g.kg^−1^); (12) total nitrogen (Nt) content (Nt: g.kg^−1^); (13) calcium carbonate (CaCO_3_) content (CaCO_3_: g.kg^−1^.); and (14) cation exchange capacity (CEC: cmol+ .kg^−1^).

#### 3.2.2 File: data_soil_pH_2023

This file contains data of soil pH, collected at the same time, from the twelve plots, in 2009 and 2020. There are six columns: (1) years of sampling (YYYY); (2) name of the cropping system (productive with high environmental performances, PHEP; no pesticide use, No-Pest; low energy consumption, L-EN; and low greenhouse gas emissions, L-GHG); (3) number of replicate (1 to 3); (4) number of plot (1 to 12); (5) soil layer (cm); and (6) pH values.

#### 3.2.3 File: data_soil_N_content_2023

This file includes data on soil nitrogen (nitrate, N-NO_3_^−^; ammonia, N-NH_4_^+^) contents, measured at a depth of 0-150 cm, collected at three different time periods (at the start and end of winter, post-harvest), in the four cropping systems, over the 2009-2020 period. There are sixteen columns: (1) year of harvest (YYYY); (2) name of cropping system (productive with high environmental performances, PHEP; no pesticide use, No-Pest; low energy consumption, L-EN; and low greenhouse gas emissions, L-GHG); (3) number of replicate (1 to 3); (4) number of plot (1 to 12); (5) number of sample (1 to 2); (6) type of the crop (cover crop, CI; main crop, CR); (7) species (for precise meanings see file “glossary_species”); (8) period of measurement (beginning of winter, BW; late winter, AW; and post-harvest, PH); (9) date of measurement (DD/MM/YYYY); (10) soil layer (0-30 cm, 30-60 cm, 60-90 cm, 90-120 cm, 120-150 cm); (11) soil bulk density, (12) soil moisture (((wet soil mass - dry soil mass)/ dry soil mass)*100); (13) soil nitrate (N-NO_3_^−^) content (kg N.ha^−1^); (14) soil ammonia (N-NH_4_^+^) content (kg N.ha^−1^); (15) total soil nitrate (N-NO_3_^−^) and soil ammonia (N-NH_4_^+^) contents (kg N.ha^−1^); and (16) percent per layer of total soil nitrate (N-NO_3_^−^) and soil ammonia (N-NH_4_^+^) contents (%).

#### 3.2.4 File: data_soil_C_N_moisture_bulk_2023

This file contains data on soil organic carbon and soil total nitrogen contents, measured in several soil layers between 0 and 40 cm depth, collected at the same time, from the productive with high environmental performances system and the low greenhouse gas emissions system, in 2009, and from all four systems in 2014 and 2020. There are fifteen columns: (1) date of sampling (DD/MM/YYYY); (2) name of the cropping system (productive with high environmental performances, PHEP; no pesticide use, No-Pest; low energy consumption, L-EN; and low greenhouse gas emissions, L-GHG); (3) number of replicate (1 to 3); (4) number of plot (1 to 12); (5) number of the sample (A to D); (6) species; (7) soil layer (0-10 cm; 10-20 cm; 20-30 cm; 30-40 cm); (8) number of soil layer (1 to 4); (9) upper soil layer limit (cm); (10) lower soil layer limit (cm); (11) soil organic carbon (C) content (C: g.kg^−1^); (12) soil total nitrogen (N) content (N: g.kg^−1^); (13) ratio soil organic carbon (C) and soil total nitrogen (N) contents; (14) residual soil moisture (% of dried soil); and (15) soil bulk density (g.cm^3^).

### 3.3 Crop management

#### 3.3.1 File: data_crop_sequence_2023

This file includes data on crop sequences, collected from the three replicates of the four cropping systems, over the 2009-2020 period. There are six columns: (1) year of harvest (YYYY); (2) name of the cropping system (productive with high environmental performances, PHEP; no pesticide use, No-Pest; low energy consumption, L-EN; and low greenhouse gas emissions, L-GHG); (3) number of replicate (1 to 3); (4) number of plot (1 to 12); (5) type of the crop (cover crop, CI; main crop, CR); and (6) species (for precise meanings see file “glossary_species”).

#### 3.3.2 File: data_agricultural_practices_2023

This file contains data relating to agronomic practices, gathered from the three replicates of the four cropping systems, over the 2009-2020 period. There are fifteen columns: (1) year of harvest (YYYY); (2) name of cropping system (productive with high environmental performances, PHEP; no pesticide use, No-Pest; low energy consumption, L-EN; and low greenhouse gas emissions, L-GHG); (3) number of replicate (1 to 3); (4) number of plot (1 to 12); (5) species (for precise meanings see file “glossary_species”); (6) date of agricultural practice (DD/MM/YYYY); (7) type of agricultural practice (for precise meanings see file “glossary_agricultural_practice_types”); (8) machine; (9) type of the crop (cover crop, CI; main crop, CR); (10) product (name of fertilizer, species (for precise meanings see file “glossary_species”), or pesticide); (11) variety; (12) quantity of products per hectare (fertilizer, seed, pesticide); (13) unit used for the quantity of product (kg, liter, or capsules); (14) quantity of nitrogen (N) (kg N.ha^−1^); and (15) dose of kernels (number of kernels per m²; number of kernel doses for maize).

### 3.4 Environmental indicators

#### 3.4.1 File: data_TFI_2023

This file contains data on the treatment frequency indexes (TFI), calculated separately for each pesticide type: herbicides (H_TFI), insecticides (I_TFI), fungicides (F_TFI), molluscicides (M_TFI), and regulators (R_TFI). Each indicator was computed on each crop sown in three cropping systems (productive with high environmental performances, PHEP; low energy consumption, L-EN; and low greenhouse gas emissions, L-GHG), over the 2009-2019 period. There was no treatment frequency indexes value in the no pesticide use system due to the ban of pesticide use in this system. There are thirteen columns: (1) year of harvest (YYYY); (2) name of the cropping system (productive with high environmental performances, PHEP; low energy consumption, L-EN; and low greenhouse gas emissions, L-GHG); (3) number of replicate (1 to 3); (4) number of plot (1 to 12); (5) species (for precise meanings see file “glossary_species”); (6) treatment frequency indexes for herbicides (H_TFI); (7) treatment frequency indexes for insecticides (I_TFI); (8) treatment frequency indexes for molluscicides (M_TFI); (9) treatment frequency indexes for insecticides minus treatment frequency indexes for molluscicides (I_TFI_net_of molluscicide); (10) treatment frequency indexes for fungicides (F_TFI); (11) treatment frequency indexes for regulators (R_TFI); (12) sum of all treatment frequency indexes (TFI_total); and (13) total treatment frequency indexes minus treatment frequency indexes for herbicides (TFI_total_net_of_herbicide).

#### 3.4.2 File: data_energy_2023

This file includes data relating to the energy consumed by the management of the cropping systems, computed from the three replicates of the four cropping systems, each year over the 2009-2019 period. There are twenty-eight columns: (1) year of harvest (YYYY); (2) name of cropping system (productive with high environmental performances, PHEP; no pesticide use, No-Pest; low energy consumption, L-EN; and low greenhouse gas emissions, L-GHG); (3) number of replicate (1 to 3); (4) number of plot (1 to 12); (5) species (for precise meanings see file “glossary_species”); (6) direct energy consumption used by all machines (E_Dir_OpCult_TT; MJ.ha^−1^); (7) indirect energy consumption for manufacturing and maintenance of all machines (E_Ind_OpCult_TT; MJ.ha^−1^); (8) direct energy consumption for plowing (E_Dir_OpCult_Plowing; MJ.ha^−1^); (9) indirect energy consumption for plow manufacturing and maintenance (E_Ind_OpCult_Plowing; MJ.ha^−1^); (10) direct energy consumption for all tillage except plowing (E_Dir_OpCult_Till; MJ.ha^−1^); (11) indirect energy consumption for tillage machine manufacturing and maintenance except plows (E_Ind_OpCult_Till; MJ.ha^−1^); (12) total energy consumption for plowing and tillage (i.e. sum of direct and indirect energy consumption for plowing and tillage; E_TT_OpCult_Plowing_Till; MJ.ha^−1^); (13) direct energy consumption used by seeders to sow main and cover crops (E_Dir_OpCult_Sowing_CR_CI; MJ.ha^−1^); (14) indirect energy consumption for seeder manufacturing and maintenance (E_Ind_OpCult_Sowing_CR_CI; MJ.ha^−1^); (15) indirect energy consumption for seed, i.e. main and cover crops, manufacturing (E_Ind_Sowing_CR_CI; MJ.ha^−1^); (16) direct energy consumption to spread fertilizers (E_Dir_OpCult_Ferti; MJ.ha^−1^); (17) indirect energy consumption for spreader manufacturing and maintenance (E_Ind_OpCult_Ferti; MJ.ha^−1^); (18) indirect energy consumption for fertilizer manufacturing (E_Ind_Ferti_product; MJ.ha^−1^); (19) direct energy consumption of pesticide sprayers (E_Dir_OpCult_Pesticides; MJ.ha^−1^); (20) indirect energy consumption for pesticide sprayer manufacturing and maintenance (E_Ind_OpCult_Pesticides; MJ.ha^−1^); (21) indirect energy consumption for pesticide manufacturing (E_Ind_Pesticides; MJ.ha^−1^); (22) direct energy consumption of herbicide sprayers (E_Dir_OpCult_Herbicide; MJ.ha^−1^); (23) indirect energy consumption for herbicide sprayer manufacturing and maintenance (E_Ind_OpCult_Herbicide; MJ.ha^−1^); (24) indirect energy consumption for herbicide manufacturing (E_Ind_Herbicides; MJ.ha^−1^); (25) direct energy consumption of mechanical weeding (E_Dir_OpCult_MecaWe; MJ.ha^−1^); (26) direct energy consumption of combine harvester (E_Dir_OpCult_Harvest; MJ.ha^−1^); (27) indirect energy consumption for combine harvester manufacturing and maintenance (E_Ind_OpCult_Harvest; MJ.ha^−1^); and (28) indirect energy consumption to dry maize kernels after harvests (E_Ind_OpCult_KeDry; MJ.ha^−1^).

### 3.5 Climate

#### 3.5.1 File: data_climatic_2023

This file contains climate data collected over the 2008-2020 period. There are six columns: (1) year (XXXX); (2) month (1 to 12); (3) day (1 to 31); (4) mean daily temperature (°C); (5) daily rainfall (mm); and (6) mean daily soil temperature at 10 cm below the surface (°C).

## 4 Experimental Design; Materials and Methods

The metadata were classified in six groups: (1) the innovative cropping systems and the field trial; (2) the plant measurements; (3) the soil properties; (4) the agricultural practices; (5) the environmental indicators; and (6) the climate.

### 4.1 The innovative cropping systems and the field trial

The four innovative cropping systems and the long-term field experiment were already widely detailed within [3,4,5,6,7]. We give the main characteristics of the innovative cropping systems and the field trial in the “Supplementary Materials” section (Figure S1).

### 4.2 Plant measurements

#### 4.2.1 Growth stages

We used growth decimal codes specific to each species:

✓ for cereals: [8,9], with some adaptations in table as follows:
  ○ to take into account the 0 before the number, e.g. for 9, we wrote x before 09 resulting in x09.
  ○ ear height measured at stem extension:
    ▪ less than 0.89 cm: tillering stage,
    ▪ 0.90 to 2.00 cm: code 30, beginning of stem extension,
    ▪ 2.01 to 2.99 cm: code C1, one node,
    ▪ 3.00 to 4.00 cm: code 32, two nodes;
✓ for faba bean (*Vicia faba L.*): [9,10];
✓ for hemp (*Cannabis sativa L.*): [11];
✓ for linseed (*Linum usitatissimum*) and camelina: [12];
✓ for maize (*Zea mays L.*): [9,10];
✓ for pea (*Pisum sativum L*.): [10,13];
✓ for winter rape (*Brassica napus L. ssp. Napus*): [9,10,14];
✓ for soybean (*Glycine max. L. MERR.*): [15].

#### 4.2.2 Glossary of growth stages

Some customizations were added for cereals, legumes, rape, and linseed.

#### 4.2.3 Plant biomass and nitrogen content

Plant biomass resulted from oven drying, at 80°C for 48 hours, of each plant sample. Due to the cost of nitrogen content analysis, two or three samples, depending on species and growth stage, were pooled and ground. A subsample was analyzed following the Dumas combustion method [16].

##### 4.2.3.1 Crops

For all crops, samples were collected at the beginning of flowering and at maturity, except for winter rape for which samples were gathered at stage 8.0 [14]. There were some supplementary collection periods for oilseed and cereal species:

✓ oilseed species (winter rape, winter linseed): aboveground parts and taproots were collected both before and after winter;
✓ cereals (barley, oat, triticale, wheat): aboveground parts were collected at the beginning of stem extension.

Depending on measurement dates and species, there were:

✓ various numbers of samples: six samples until flowering stage, and nine to twelve samples at maturity. At maturity, samples were also used for yield component measurements. There are some peculiarities of samples. At maturity, due to the high aboveground biomass for maize every year, except in 2009, each sample was divided into two subsamples (called A and B). For the same reason, in 2010, the samples were halved for the productive with high environmental performances system (plot 10; sample 2), for the low energy consumption system (plot 12; samples 7 and 8), and for the low greenhouse gas emissions system (plot 6; sample 4). The second subsample was called “_bis”;
✓ various sizes of samples: two adjacent rows of one meter resulted in various size samples (0.25 m² to 3 m²) depending on the seeder machine (see metadata of agricultural practices, Table 1). There was a peculiarity in 2009: 5.22 m² for the maize sample in the no pesticide use system. The sampling method used at maturity is provided in the section “Yield components”.

**Table 1:** Characteristics of seeder machines.

Seeder machine	Inter raw length(cm)	Surface (m²) of 2 adjacent rows of 1 meter
Amazone: seed drill machine combined with a rotary harrow	12.5	0.25
Semeato: direct seeder machine	17.0	0.34
Kuhn: direct seeder machine for cereals in the no pesticide use system	24.0 and 8.5 alternately	0.33
Nodet: spaced planter for maize	80.0	1.60

For all crops, at maturity, kernels were separated from the vegetative parts of the plant, i.e. straws and pod walls for legumes (stems_pods), straws and rachis for cereals (straws_rachis_ears), straws and panicles for oat (straws_panicles), and stalks and cobs for maize (stalks_cobs). For rape, the different aboveground parts (stems, pods and green seeds) were pooled.

##### 4.2.3.2 Cover crops, volunteers, and weeds

Six samples of 1 m² were taken in the autumn (from mid-November to mid-December). Sometimes, each species of the cover crop mixture was weighted separately. As we were required to simulate soil carbon sequestration, some estimations were provided for missing data: in 2009 for plots 1, 2, 5, 8, and 9; in 2010 for plots 7, 8, and 10; in 2011 for plots 4, 8, 9, and 10; in 2012 for plot 12.

Depending on their growth, we also collected volunteers and weeds with the same method, i.e. six samples of 1m² each.

#### 4.2.4 Yield components

For all species except maize, and due to the difficulties of separating the plants at maturity, the number of plants were counted early using samples dedicated to the biomass measurements:

✓ for cereals: at the beginning of tillering (stage BBCH 21 [8,9] i.e. mid-March) or over the spring,
✓ for legumes: at the beginning of flowering or at maturity (stages BBCH 61 and 92 respectively [9,10,13]),
✓ for linseed: at the end of winter or beginning of spring), based on 6 to 7 samples.

Sometime, the number of plants were managed at maturity, but with less accuracy.

For maize, the number of plants were counted at maturity (stage BBCH 92 [9,10]). In 2019, there was one supplementary counting in June.

For the other yield components, nine to twelve samples were collected at maturity (stage BBCH 92 [10]), and three more samples (1_0N, 2_0N, 3_0N) were harvested in a specific non-fertilized area. These samples were also used to measure aboveground biomass and N content. For winter rape, counting was done at stage 8.0 [14] to avoid losses of dried pods which occurred at maturity. Area sizes were two adjacent rows of one meter for cereals and legumes, two adjacent rows of 2.5 meters for maize, and 0.5 m² and 1 m² for linseed and winter rape respectively. Surface areas varied according to the seeder machine (see metadata on agricultural practices in Table 1). All plants (aboveground parts and roots, or taproots for winter rape) were collected from the field trial, except for maize for which only aerial parts were harvested. In laboratory, roots were removed from the samples, and number of branches were counted.

For all species, except for winter rape, reproductive and vegetative parts were separated (see metadata on “Aboveground biomass and N content”). Depending on the species, either a subsample (linseed, cereals) or the whole sample of each kernel sample was used to calculate the thousand-kernels weight (gram; 0% of dry matter).

Depending on the species, there were some specific counting.

For linseed, we counted: (1) the number of fertile branches (with capsules with at least one kernel) and sterile branches (without capsule, or with capsules without kernel); and (2) the number of fertile capsules (with kernels) and sterile capsules (without kernel).

For cereals, at the beginning of tillering, from each sample a subsample of 20 plants was used to count number of tillers with more than three sub-tillers. There were some sample peculiarities as follows: (1) in 2009, there were 12 samples for spring oat (plot 5); (2) in 2009, due to take-all disease on winter wheat (plot 11), three samples (1_PE, 2_PE, 3_PE) were collected from the diseased area; (3) in 2010, due to the large plant biomasses of winter wheat, two subsamples were collected and called “_bis” (plot 6, sample 4; plot 10, sample 2; plot 12, samples 7 and 8); and (4) in 2020, due to growth problems for winter wheat (plots 4, 8 and 12), spring barley (plot 7), and triticale (plots 2 and 9), no yield components were provided, except for the total number of plants. For spring barley, there were nine samples.

For legumes, the number of fertile pods (with kernels) and the number of sterile pods (without kernel) were counted.

For maize, the specific counting were as follows: (1) the number of fertile cobs (with kernels); (2) the number of all cobs (fertile and sterile cobs); (3) the average number of rows per cob, resulting from the mean of the row numbers counted from all the cobs of the sample. In 2009, samples 13, 14, and 15 corresponded to an area where there was a second sowing.

#### 4.2.5 Yield harvested with a combine harvester

Yield. Six samples per plot were collected, each year, at maturity, with a combine harvester. Sample surfaces ranged from 75 m² to 140 m², depending on the harvester (i.e. widths of the cutter bar were 1.5 m for linseed, 4 m for maize, and 3 m for all other crops), and the length of the harvested plot (i.e. to avoid border effects we excluded 6 to 8 m on each side). Each kernel sample was weighted separately. From each sample, a subsample of almost 500 grams was quickly collected to measure the moisture (% of dry matter; the subsamples were oven-dried at 80°C for 48 hours). Yield values (q. ha^−1^, 0% of dry matter for all species, except for hemp) were calculated as the ratio between the kernel weight and the surface harvested.

Thousand-kernels weight. After the drying process of each kernel sample, a subsample of 70-80 grams was used to calculate the thousand-kernels weight (expressed in grams).

For hemp, different harvesting methods were used, depending on the combine harvester. In 2011, 2012 and 2013, only straws were harvested, while both straws and grains were harvested separately in 2017 and 2019. Yields were expressed for kernels in q.ha^−1^ (0% of dry matter), and for straws in ton (t) dry matter.ha^−1^ (0% of dry matter). Data were available in both files: “File: data_plant_biomass_nitrogen_content_2023”, and “File: data_yield_combine_all_species_2023”.

Particularity of soybean: due to the manual harvest in 2014, the twelve samples had a lower size than the other (almost 39 m²). No grain moisture was measured.

In 2011 and 2012, there were 10 samples for the linseed plot.

### 4.3 Soil properties

#### 4.3.1 Structural and chemical soil properties

Measurements were managed just before the beginning of the trial implementation in 2009. Each plot was divided into four sub-plots. In each sub-plot seven soil samples, well balanced out over the sub-plot, were collected manually with an auger (layer 0-25 cm). Values are expressed considering an air-dried soil. Five textural fractions, i.e. clay (g.kg^−1^), coarse silt (g.kg^−1^), fine silt (g.kg^−1^), coarse sand (g.kg^−1^), and fine sand (g.kg^−1^), were measured without decarbonation (NF X 31-107). Chemical properties were also measured: organic and mineral carbon (C) contents (C: g.kg^−1^; NF ISO 10694), total nitrogen (N) content (N: g.kg^−1^; NF ISO 13878), calcium carbonate (CaCO_3_) content (CaCO_3_: g.kg^−1^; NF ISO 10693), and cation exchange capacity (CEC; cmol+.kg^−1^; Metson method, NF ISO 31-130).

#### 4.3.2 Soil pH

Soil samples were collected according to the same method as that used in 2009 and 2020 to measure soil structural properties. The pH data were measured according to the NF ISO 10390 process (pH in water).

#### 4.3.3 Soil nitrogen (nitrate and ammonia) content

Measurements were collected at three different periods over the year: at the beginning of winter around November 15; in late winter around February 15; and about eight days post harvesting of the main crop. In each plot, six soil samples were collected manually with an auger and stored in a cold box (4°C) until analysis. Five layers were measured: 0-30 cm, 30-60 cm, 60-90 cm, 90-120 cm, and 120-150 cm. Three samples from each layer were pooled to generate two soil samples per layer for each plot. Water content was measured gravimetrically, according to the international standard method (NF ISO 11465). Analysis of nitrate (N-NO_3_^−^) and ammonia (N-NH_4_^+^) contents were precisely described in [7], according to the international standard method (NF ISO 14255). Results were expressed in kg N per hectare.

#### 4.3.4 Soil organic carbon content, soil total nitrogen content, bulk density, and residual soil moisture

The measurements were carried out on each replicate plot of the four cropping systems, in 2014 and 2020, for four layers (∼0-10 cm, ∼10-20 cm, ∼20-30 cm and ∼30-40 cm). Because the cropping practices and the crop sequence prior to 2009 (the year of the trial implementation) were homogeneous across the whole field trial, only half of the plots (i.e. the productive with high environmental performances, and low greenhouse gas emissions plots) were analyzed in 2009. Same method was applied throughout the study.

Soil organic carbon and soil total nitrogen contents. Each plot was divided into four sub-plots. In each sub-plot, three soil samples, well balanced over the sub-plot, were collected manually using a percussion corer with a 5.5 cm diameter. The three subsamples were pooled, and the soil organic carbon contents were measured using the NF ISO 10694 process. The organic carbon content was calculated as the difference between the total carbon content and the mineral carbon (Cmin) content obtain from the calcium carbonate content (Cmin = 0.12 x CaCO_3_). Values are expressed considering an air-dried soil, but residual soil moisture is also provided.

Note: several outlier data of soil carbon content were modified. Some data were computed (row 26: original value was 22.5; row 46: original value was 17.1) and some samples were re-analyzed (rows 323, 324 and 325).

The soil total nitrogen contents were analyzed according the Dumas method (NF ISO 13878).

Bulk density and residual soil moisture. Each plot was divided into four sub-plots. One soil sample was collected on each sub-plot using a steel cylinder of 98 cm^3^ inserted vertically in the soil at each of the four layers. Soil was weighed after drying for 48 hours at 105°C. Soil moisture was measured gravimetrically, according to the international standard method (NF ISO 11465).

### 4.4 Agricultural practices and indicators

#### 4.4.1 Agricultural practices

All agricultural practices were recorded continuously over the twelve-year period. The crop sequences, and the species sown in each replicate of each system over the 2009-2020 period are detailed in the file named: data_crop_sequence_2023 (for precise meanings see file “glossary_species”).

Details of agricultural practices

For hemp, harvest operation includes cutting and straw swathing.

The direct seeder machine had two hoppers that allowed the sowing of two species simultaneously, one sowing and one nitrogen fertilizer spreading, or one sowing and one molluscicide spreading.

Descriptions were given for: (1) the corresponding surface of two adjacent rows of one meter for different seeders (Table 1) required to compute the amounts of aboveground biomasses and the quantity of nitrogen produced per hectare; (2) the thousand-kernels weight of different species (Table 2) needed to calculate the indirect energy consumption for cover crop seeding operations; and (3) the depth of soil tillage (Table 3) used to calculate soil carbon sequestration.

**Table 2:** Thousand-kernels weight (gram) of different species.

Species	Thousand-kernels weight (gram)
Barley (spring)	51
Buckwheat	23 (average of 12 and 35)
Clover (Alexandry)	2.9
Clover (white)	0.7 (average of 0.65 and 0.80)
Clover (other varieties)	2.3 (average of 1.8 and 2.8)
Faba bean (spring)	460
Hemp	17
Lentil	150
Linseed (winter)	6.6
Mustard	5
Oat (spring)	50
Pea (spring)	253
Rape (winter)	4.2
Soybean	149
Vetch	60

**Table 3:** Depth of soil tillage.

Machine	Depth of soil tillage (cm)
Hoe	7
Crusher	3
Disc harrow	8
Vibrating tine cultivator	10
Rotary harrow	7
Plough	25
Rotavator (scalper)	0
Pressing roller	0

#### 4.4.2 Treatment frequency index

The treatment frequency indexes were computed according to [1]. The recommended doses, required for the calculations, were found at https://alim.agriculture.gouv.fr/ift/doses-reference/2018. These references were regularly updated to take into account the pesticide market withdrawal. Seed treatments were excluded from the treatment frequency index computations.

#### 4.4.3 Energy consumption

The energy consumption (MJ.ha^−1^) was computed annually from each plot, based on [2,17], and [18]. Direct energy consumptions included fuel, lubricants and electricity used to power farm machines and tractors. For farm machines, we took into account tractor power, width of the machine or number of ploughshares, and working hours. Indirect energy consumption resulted from the energy used in the manufacture, formulation, packaging and maintenance of inputs, such as machines, seeds, fertilizers and pesticides. We used specific pesticide energy coefficients for computations: 0.204, 0.295, and 0.282 MJ per gram of active ingredient for fungicides, herbicides, and molluscicides respectively (Table 4). When straws were removed from the plots, energy computation took into account only swathing operations. Fossil fuel used for input transportation from the manufacturing site to the trial were not included in the computations.

**Table 4:** Energy (direct and indirect) inputs, and main products (fertilizers, pesticides, and seeds) of agricultural practices. Consumptions, based on [2] took into account: (1) for farm machinery: tractor power, width of the machine or number of ploughshares, and the working hours; and (2) for pesticides: active ingredient quantities (g.kg^−1^ or g.l^−1^), and specific pesticide energy coefficients (0.204, 0.295, and 0.282 MJ per gram of active ingredient for fungicides, herbicides, and molluscicides respectively).

			Direct energy input (MJ.ha^−1^)	Indirect energy input (MJ.ha^−1^)
Agricultural operations	Tillage	Ploughing: five ploughshares; tractor 150CV; 200 ha.hour^−1^	1260	91.0
		Deep cultivator: tractor 150CV; width: 4.5m; 200 ha.hour^−1^	1047	73.0
		Shallow cultivator: tractor 130CV; width 6m; 120 ha.hour^−1^	441	84.0
		Rotary harrow: tractor 130CV; width 4m; 80-150 ha.hour^−1^	672	90.0
		Crushing (maize stalk, cover crop): tractor 130CV; width 4m; 150 ha.hour^−1^	403	59.0
		Roller: tractor 130CV; width 9m; 125 ha.hour^−1^	157	118.0
	Seedling	Seed drill combination with harrow: tractor 160CV; width 4m; 120 ha.hour^−1^	827	109.0
		Direct seeder: tractor 120CV; width 3m; 120 ha.hour^−1^	279	125.0
		Spaced planter (maize): tractor 70CV; width 6 rows; 3 ha.hour^−1^	226	51.0
	Fertilization	Spreader: tractor 130CV; width 24m; 100 ha.hour^−1^	126	14.0
	Mechanical weeding	Hoes: tractor 70CV; width 4m; 80 ha.hour^−1^	226	46.0
		Spiked harrow: tractor 100CV; width 12m	61	30.0
	Spraying	Pesticides: tractor 90CV; 3400 l; 200 ha.hour^−1^	65	17.0
	Harvest	Combine harvester (all crops except hemp): 260CV	1512	173.0
		Mowing (hemp): tractor 75CV; width 2m	509	430.0
		Swathing (hemp): tractor 100CV; width 6.5m	162	98.0
Fertilizers		Nitrogen: ammonitrate 33.5%		15.9
		Phosphate: super 45		4.4
Pesticides	Herbicides	Roundup max 480 (glyphosate): 3-4 l.ha^−1^		141.6
		Bofix (winter wheat): 3 l.ha^−1^		76.7
		Colzor trio (winter rape): 4 l.ha^−1^		119.5
	Molluscicides	Mesurol pro: 3 kg.ha^−1^		5.6
		Metarex ino: 5 kg.ha^−1^		11.5
	Fungicides	Amistar (winter wheat): 1 l.ha^−1^		102.0
		Caramba star (winter wheat): 1 l.ha^−1^		18.4
Seeds		Cereals: oats, barley, winter wheat, triticale		3.0 to 4.0
		Oilseeds: winter rape, winter flax		8.0 to 8.5
		Maize		12.3
		Legumes: pea; faba bean		2.6
		Hemp		10.1
Kernel drying				3.45 GJ.t H_2_O^−1^

### 4.5 Climate

The data were collected from an automated INRAE meteorological station (no. 78615002: latitude 48.838°N, longitude 1.953°E, elevation: 125 m), located 150 m from the trial.

## Limitations

Due to the COVID pandemic, no agriculture practices or measurements were recorded in spring 2020. It would therefore be difficult to include this year within plant growth model simulations.

Several measurements of pests (insects, diseases, and weeds) were managed. However, due to the workload it requires, they were collected in an irregular manner over the twelve years.

## Conclusion

All these data have been used in others papers to describe the agricultural practices of the innovative cropping systems in order (1) to compare the new and the current cropping systems [3,4,5], and [6]; to assess the ability of the four innovative systems to meet the constraints and goals [4]; to quantify the yield in a pesticide free cropping system and the associated environmental impacts [5]; to quantify the production of a very low-energy cropping system and its environmental performances [6]; and to identify how these performances were reached (agronomic diagnostics were included in each paper), and the technical lock-ins that still exist.

## Ethics Statement

The authors have read and followed the ethical requirements for publication in *Data in Brief* and hereby confirm that the current work does not involve human subjects, animal experiments, or any data collected from social media platforms.

## CRediT authorship contribution statement

**Caroline Colnenne-David:** Conceptualization, Methodology, Validation, Formal analysis, Resources, Writing – original draft, Writing – review & editing, Project administration, Funding acquisition, Supervision. **Marie-Hélène Jeuffroy:** Conceptualization, Writing – original draft, Funding acquisition, Supervision. **Gilles Grandeau:** Resources. **Fabien Ferchaud:** Resources. **Thierry Doré:** Conceptualization, Writing – original draft, Funding acquisition, Supervision.

## Data Availability

Innovative cropping systems designed to reach both environmental and production targets: data set of biotic and abiotic variables from a twelve-year French field trial (Original data) (Open Research Data Portal at INRAE).

## Appendix Supplementary materials

Image, application 1
