# Supplementary material for: Innovative cropping systems designed to reach both environmental and production targets: Data set of biotic and abiotic variables from a twelve-year French field trial
Source: Data Brief. 2024 Apr 5;54:110398. doi: 10.1016/j.dib.2024.110398 (PMC11043882; doi:10.1016/j.dib.2024.110398)
Supplement: Supplementary file 1 [file mmc1.docx]

**Supplementary Materials SM1**

**Description of the four innovative cropping systems**

The Productive with High Environmental Performances (PHEP) system was designed to lessen environmental impact: (i) pesticide uses were reduced by enhancing crop diversity, lengthening the crop sequence, and including highly resistant varieties and species; (ii) energy consumption was decreased by minimizing plowing to only once over the five-year crop sequence, and by lowering nitrogen (N) fertilizer amounts resulting from legume sowing within the crop sequence; and (iii) nitrate losses could be met by sowing cover crops before each spring species. This cropping system, also designed to achieve the maximum yield given the environmental targets, was used as the reference system for comparisons with the other systems.

The No-Pesticide (No-Pest) system was designed to meet an additional environmental constraint: no pesticides, not even those used within organic systems, were allowed, while chemical fertilizers could be used. The crop sequence was lengthened, involving a wide range of resistant species; plowing, tillage and mechanical weeding were managed to destroy weeds; and yield targets were reduced compared to the PHEP system.

In the low energy (L-EN) system, a decrease of both direct and indirect energy consumption was targeted: (1) plowing and tillage were banned, and used direct sowing; (2) the amount of N fertilizer was reduced by sowing many legumes and high-efficiency N use species, and yield targets were reduced.

In the low greenhouse gas (L-GHG) system, to mitigate GHG emissions, high soil carbon sequestration was targeted (i.e. neither plowing nor tillage were involved; large amounts of residues were provided), and nitrous oxide emissions were reduced by decreasing the amount of mineral N fertilizer.

**Figure S1.** Experimental design with the four cropping systems, and the three replicates, located at the AgroParisTech experimental farm (France, N 48.84°, E 1.95°). The crops sown at the beginning of the field trial assessment (2009) are indicated on each plot. Cropping systems: PHEP (productive with high environmental performances), No-Pest (no pesticide use), L-EN (low energy consumption), L-GHG (low greenhouse gas emissions). w and s indicate winter and spring crops, respectively.

L-GHG

L-EN

PHEP

No-Pest

L-GHG

PHEP

No-Pest

PHEP

L-EN

L-GHG

No-Pest

L-EN

65m

63m

Replicate 3

Replicate 2

Replicate 1
